# Neural mechanisms in resolving prior and likelihood uncertainty in scene recognition

**DOI:** 10.1016/j.isci.2025.112663

**Published:** 2025-05-13

**Authors:** Kojiro Hayashi, Risa Katayama, Keisuke Fujimoto, Wako Yoshida, Shin Ishii

**Affiliations:** 1 Graduate School of Informatics, Kyoto University, Kyoto 606-8501, Japan; 2 Department of AI-Brain Integration, Advanced Telecommunications Research Institute International, Kyoto 619-0288, Japan; 3 Nuffield Department of Clinical Neurosciences, University of Oxford, OX3 9DU Oxford, UK; 4 Department of Neural Computation for Decision-making, Advanced Telecommunications Research Institute International, Kyoto 619-0288, Japan; 5 International Research Center for Neurointelligence, the University of Tokyo, Tokyo 113-0033, Japan

**Keywords:** Neuroscience, Sensory neuroscience, Cognitive neuroscience

## Abstract

Recognizing real-world scenes requires integrating sensory (likelihood) and prior information, yet how the brain represents these components remains unclear. To investigate this, we employed deep image transformation to generate images with parametrically controlled naturalness, enabling precise manipulation of likelihood uncertainty. Concurrently, we designed a sequential image-scene recognition task that quantitatively modulates prior information. By combining these AI-generated images with the task, we conducted a functional magnetic resonance imaging (fMRI) experiment enabling systematic control of both likelihood and prior information. The results revealed that higher visual areas were activated when viewing images with low likelihood uncertainty. In contrast, the default mode network, which includes the medial prefrontal gyrus, inferior parietal lobule, and middle temporal gyrus, exhibited higher activation when more prior information was available. This approach highlights how applying AI technology to neuroscience questions can enhance our understanding of neural mechanisms underlying scene recognition.

## Highlights

• Images with varying levels of naturalness were generated using deep learning
• Tasks involving these images manipulated the uncertainty of both likelihood and prior
• Human scene recognition performance was modeled within a Bayesian framework
• Brain regions representing likelihood and prior information were identified

## Graphical abstract

**Figure:**
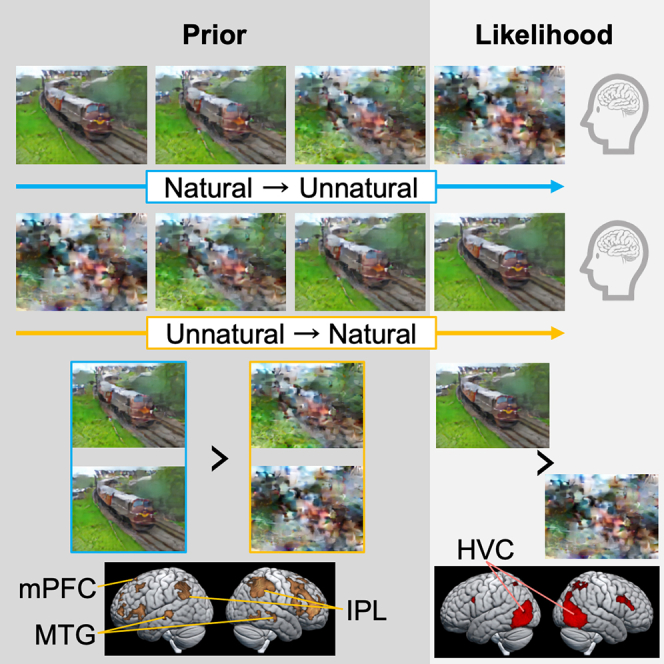

## Introduction

Our world is inevitably uncertain due to the limitations of our knowledge and available sensors. Humans, too, often find themselves in situations where they lack a clear understanding of their surroundings. Imagine being presented with several photographs of a particular location, each containing incomplete information due to deterioration or partial photography. If the scenery is familiar, the viewer can quickly recognize it. However, if the scenery is unfamiliar, more photographs are needed to provide clues. Moreover, having information about the landscape of a photo in advance makes it easier to recognize the location. Thus, the recognition of location, or more broadly, scenes, requires the integration of prior knowledge and multiple cues. Recognition through such integration exhibits different aspects depending on the presence or absence of prior knowledge and its quantity. In this study, we investigated the computational processes of the brain that lead to recognition, focusing specifically on “scene recognition”. Our emphasis was on the process of recognizing a scene by integrating prior knowledge (as demonstrated in the above example with “knowledge about the landscape”) and incomplete observations (“multiple incomplete pictures of the scenery”).

According to the “Bayesian brain” hypothesis,^1^^,^^2^ perception is generated by the brain through the integration of sensory information with its internal model of the external world, referred to as the “world model”. This integration process leads to recognition, prediction, and decision-making. The hierarchical nature of the visual system has long been established, where lower-order areas transmit prediction errors to higher-order areas, while higher-order areas provide feedback to lower-order areas based on their predictions.^3^^,^^4^ The “Bayesian coding hypothesis”^2^^,^^5^^,^^6^^,^^7^^,^^8^ proposes that the brain encodes states and predicts them using probability distributions or approximations. Previous studies indicate that Bayesian inference in human perception computations involves multiple sensory inputs and prior information, as observed in phenomena like motion illusions,^9^ sensorimotor learning,^10^ and cue integration.^11^ Several investigations^12^^,^^13^ have demonstrated that human scene recognition can be effectively modeled using Bayesian inference grounded in structured probability distributions.^14^ Nevertheless, the representation of uncertainty regarding sensory and prior information in the brain remains unclear.

Studies on brain activity during object recognition^15^^,^^16^ have indicated that the availability of prior knowledge influences the neural representation of the frontoparietal network (FPN) and default mode network (DMN) when resolving sensory stimulus ambiguity. Various studies^17^^,^^18^^,^^19^ have also demonstrated that the presence or absence of prior information affects neural activity in visual areas when resolving the ambiguity of visual stimuli. Moreover, Visalli et al.^20^ have proposed that the FPN plays a role in temporal expectation updating and surprise. Despite reports of differences in brain activity related to uncertainty about prior information, the specific functions of the DMN and FPN in processing prior information and the changes in brain activity during the gradual resolution of ambiguity remain incompletely understood. Furthermore, visual recognition tasks in these studies commonly employed simple visual stimuli, typically black and white images. Some studies^21^^,^^22^ suggested that color information influences object recognition and neural activity. This raises questions about the generalizability of the results to natural situations and how visual information is processed in complex real-world environments.^23^ To investigate whether Bayesian scene recognition occurs in natural situations, it is valuable to use stimuli whose naturalness can be objectively evaluated.

To elucidate the neural representations associated with the likelihood and prior uncertainty in scene recognition, we conducted a functional magnetic resonance imaging (fMRI) experiment. This experiment utilized images with parametrically varied scene naturalness (i.e., scene recognizability), achieved through an artificial image generation method.^24^ In our scene recognition task, we systematically manipulated prior and sensory information by presenting images in a sequence that gradually increased or decreased their naturalness. Adding noise or scrambled images can be considered a method for manipulating the context of natural images. However, there is a risk that the saliency map,^25^ which highlights the regions of an image that are most likely to attract attention, may also change. It has been reported that the magnitude of salience^26^ and the most salient position^27^ in natural images are encoded in the brain, so changes in the saliency map can affect brain activity. Therefore, in this study, we used the image transformation method,^24^ which reduces the naturalness of the image while maintaining the saliency map of the natural image.

We propose a Bayesian model that updates the probability of recognizable through Bayesian formulas, successfully elucidating participants’ scene recognition behaviors. Employing this Bayesian model, we analyzed brain imaging data and discovered the involvement of higher visual areas in resolving sensory (likelihood) uncertainty. Additionally, the DMN regions were implicated in representing prior uncertainty. Furthermore, the activation of the FPN regions coincided with participants altering their behavior, suggesting a potential role of the FPN in Bayesian surprise.

## Results

### Image-scene recognition task

**Figure 1:**
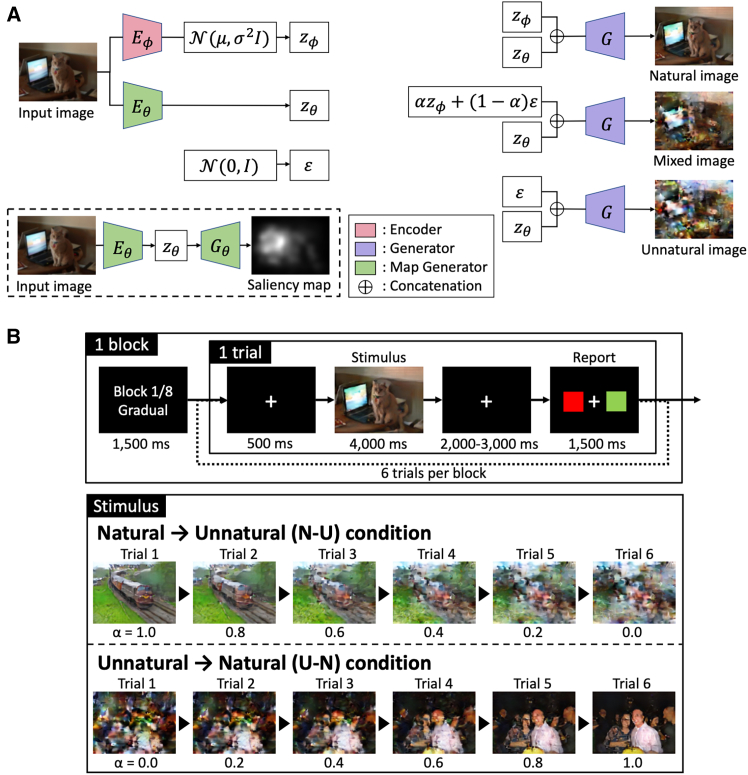
Experimental procedure (A) Architecture of image generation. One set of images covered “natural,” “mixed,” and “unnatural” images with different levels of naturalness (expressed as a scalar α), artificially generated by a deep learning technology (GANSID). Our image-generation method includes an encoder, generator, and map generator. The generator, trained with the latent variable $Z_{\phi }$ from the input image and the map latent variable $Z_{\theta }$, recreates the original image. The map generator was pre-trained using Itti’s saliency map.^28^ These images are produced using $Z_{\theta }$ and a mix of $Z_{\phi }$ and a random normal variable ε at ratio α. By varying α from 0 to 1, images with different naturalness were generated. The natural and unnatural images were characterized by α = 1.0 and α = 0.0, respectively, and the mixed images by in-between α values, α = 0.2, 0.4, 0.6, and 0.8. (B) Procedure for a single block consisting of six trials. The participants were first instructed on the block condition (Binary or Gradual). In a Binary block, six images including natural (α = 1.0) and unnatural (α = 0.0) images, which were generated from different original images, were presented in random order (not shown in the figure). In a Gradual block, a set of six images generated from one original image were presented in two different orders; in the N-U condition, the images were displayed in the order of decreasing naturalness, i.e., from natural (α = 1.0, N) to unnatural (α = 0.0, U), whereas in the U-N condition, they were displayed in the order of increasing naturalness, i.e., from unnatural to natural. In each trial, participants were presented one image (for 4,000 ms) and were required to report whether they could recognize the image scene or not by selecting a green (corresponding to “Yes,” that is, recognizable) or red square (corresponding to “No,” or un-recognizable) using an MRI-compatible response box (within 1,500 ms).

In each trial of the scene recognition task, participants were required to examine a single image and indicate whether they could identify the scene depicted in the image. The images were generated from natural scene images by systematically adjusting the level of naturalness, denoted as α (STAR Methods and Figure 1A). To generate these images, we modified “Generative Adversarial Network with Maintained Saliency for Image Deconstruction (GANSID^24^)” technique. The variations included a natural image (with α = 1.0), an unnatural image (α = 0.0), and mixed images with α values ranging from 0.2 to 0.8 (Figure 1A). Each block comprised six consecutive trials featuring images derived from the same original image. There were two task conditions: one where the natural image was initially presented and gradually lost its naturalness (natural to unnatural; N-U condition) and another where the unnatural image gradually transformed into a natural one (unnatural to natural; U-N condition) (Figure 1B). As a control condition, we administered a block in which six natural or unnatural images were randomly presented.

### Recognition patterns and Bayesian model

**Figure 2:**
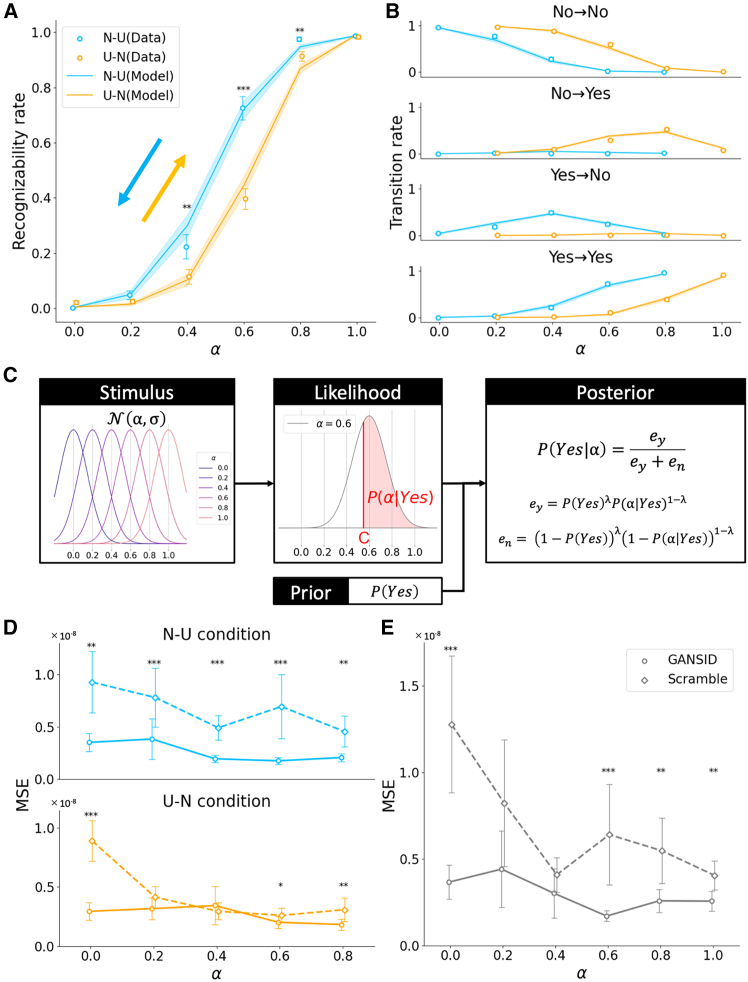
Behavioral results and model of participants’ scene recognition (A) Participant-wise image scene recognition rate as a function of the naturalness level of the image under the N-U (blue) and U-N conditions (orange) (*N* = 30). The circles and solid lines represent the averages of the actual data and model predictions (see (C)) across the participants, respectively. The error bars and shaded areas represent the standard error. For each naturalness level, the recognition rate was statistically compared between the N-U and U-N conditions (Wilcoxon signed-rank test; ∗∗*p* < 0.01, ∗∗∗*p* < 0.001, Bonferroni-corrected). (B) The proportion of the trials categorized by four different response patterns in the previous and current trials (No→No, No→Yes, Yes→No, and Yes→Yes). For example, No→Yes indicates the trials in which the participants evaluated the image in the current trial as recognizable and that in the previous trial as un-recognizable, in other words, they switched their response. The circles and solid lines represent the means of the participants and model predictions (see (C)), respectively. The error bars and shaded areas represent the standard error. (C) A Bayesian model of participant scene recognition. This model assumes that when the participants observed the image with the naturalness level α, the probability that they recognized the scene of the image followed a normal distribution N(α,σ). For each participant, threshold C was estimated to be common to all alpha values, with the area to the right side of the threshold defined as P(α|Yes). P(α|Yes) denotes the probability or likelihood of recognition of the scene provided by the current image only. Finally, the posterior probability P(Yes|α) that the participant responded “Yes” to the current image was updated according to Bayesian formula. (D) The mean squared error (MSE) between the fixation density maps (FDMs) with the natural images (α = 1.0) and those for images with the other naturalness-level (α) images in the N-U (blue) and U-N conditions (orange) (*N* = 20). The circles and solid lines represent the averages across all participants for the GANSID images, while the diamonds and dashed lines represent the means for the scrambled images. The error bars indicate the standard error (Wilcoxon signed-rank test; ∗*p* < 0.05, ∗∗*p* < 0.01, ∗∗∗*p* < 0.001, Bonferroni-corrected). (E) The MSE of the FDMs for each naturalness-level image between the N-U and U-N conditions. The format is the same as (D).

In both the N-U and U-N conditions, the participants tended to report that images with higher levels of naturalness were more recognizable (Figure 2A). In a comparison of the two conditions, for images with a medium degree of naturalness, the frequency of responses as recognizable was significantly higher in the N-U condition than in the U-N condition (Wilcoxon signed-rank test with Bonferroni correction, *p* = 2.3 × 10^−1^, 5.4 × 10^−1^, 8.4 × 10^−3^, 1.9 × 10^−5^, 7.8 × 10^−3^, 1.0 for images with α = 0.0, 0.2, 0.4, 0.6, 0.8, 1.0, respectively; Figure 2A). The timing of the reversal of recognition also varied between conditions: in the N-U condition, the reversal of recognition was more frequent in the middle of the block (α = 0.4), whereas, in the U-N condition, the reversal of recognition was more frequent toward the end of the block (α = 0.8) (Figure 2B).

We proposed a Bayesian model to explain participants’ image-scene recognition performance (Figure 2C). In this model, the probability that the participant responded “Yes” to the current image is updated using the Bayesian formula. When updating the probability, the hyperparameter λ takes a balance between the likelihood and prior information. Participants’ recognition behaviors were assumed to be influenced by two hyperparameters: the propensity to respond as recognizable (C) and the persistence of the prior information for recognizability of the image scene (λ). We compared four models with various settings of these hyperparameters (see STAR Methods) and confirmed that the model with the two parameters determined from the behaviors best reproduced the participants’ behaviors (C = 0.55 ± 0.08, λ = 0.23 ± 0.11; mean ± standard deviation) based on Bayesian Information Criterion (BIC) scores (Table S1). This model successfully reproduced the participants’ responses for recognizability (Figure 2A, solid lines; Figure S1) and timing of the reversal of recognition (Figure 2B, solid lines).

### GANSID images controlled visual attention

To confirm that our GANSID images controlled the participants’ bottom-up visual attention, we conducted eye movement measurements (please refer to STAR Methods, eye tracking task). We obtained the fixation density map (FDM) by applying kernel density estimation using non-isotropic Gaussian kernels to the histogram of eye gaze points of each participant as he or she viewed the images and then calculated the mean squared error (MSE) between the FDM of natural images (α = 1.0) and images with other α in the N-U and U-N conditions. The results showed that GANSID images had smaller MSE compared to scrambled images (Wilcoxon signed-rank test with Bonferroni correction, *p* = 1.0 × 10^−3^, 9.5 × 10^−5^, 4.8 × 10^−5^, 2.9 × 10^−5^, 2.0 × 10^−3^ for images with α = 0.0, 0.2, 0.4, 0.6, 0.8, respectively in N-U condition, *p* = 1.9 × 10^−5^, 2.9 × 10^−1^, 1.0, 4.2 × 10^−1^, 3.5 × 10^−3^ in the U-N condition; Figure 2D). We also calculated the MSE between the N-U and U-N conditions of the FDM for the same images, and found that the GANSID images did not show large differences in fixation points between the U-N and N-U conditions compared to scrambled images (Wilcoxon signed-rank test with Bonferroni correction, *p* = 8.0 × 10^−5^, 1.0 × 10^−1^, 6.4 × 10^−2^, 5.7 × 10^−5^, 1.9 × 10^−3^, 7.3 × 10^−3^ for images with α = 0.0, 0.2, 0.4, 0.6, 0.8, 1.0, respectively; Figure 2E).

### Visual areas responded differently to sensory information

**Figure 3:**
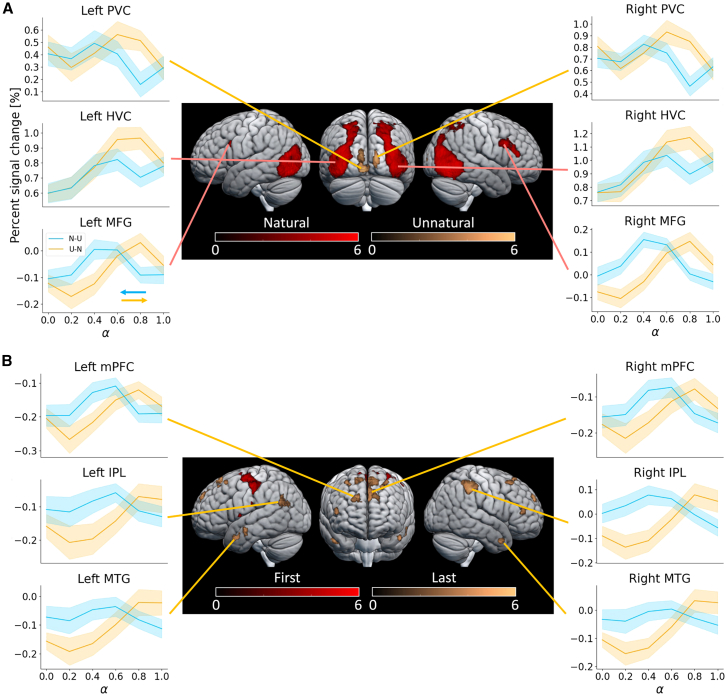
Brain activity induced by the image naturalness and the image presentation timing Brain activation areas reflecting the main effect of naturalness (A, red) and unnaturalness (A, yellow) and whether the image was presented in the first (B, red) or the last trial (B, yellow) in the block were examined using two-way ANOVA (voxel level: *p* < 0.0001, uncorrected, cluster level: *p* < 0.05, FWE-corrected). No statistically significant interaction effects were observed. The line plots show the percent signal change (PSC) in each anatomical ROI (see STAR Methods) as a function of the naturalness level α of the presented images in the N-U (blue) and U-N conditions (orange), respectively. Solid lines indicate the average values across the participants. The shaded area represents the standard error.

To analyze the brain images, we conducted a 2 × 2 ANOVA, considering naturalness (natural or unnatural image) and the timing of presentation in the block sequence (first or last trial) as factors (Figure 3; Table S2). When natural images were presented, activation was observed in bilateral middle frontal gyri (MFG, Broadmann areas (BAs) 9/46) and bilateral higher visual cortices (HVC), including extrastriate cortices, superior parietal lobules (SPL, BA 7), fusiform gyri (BA 37), and posterior parahippocampal gyri (left MFG: MNI coordinate = [-44,14,28], right MFG: [42,14,30], left HVC: [-38,-82,20], right HVC: [46,-68,-12], Figure 3A, red). Conversely, only the primary visual cortex (PVC, BA 17) showed activation when unnatural images were presented (left: [-2,-90,-10], right: [14,-96,10], Figure 3A, yellow).

**Figure 4:**
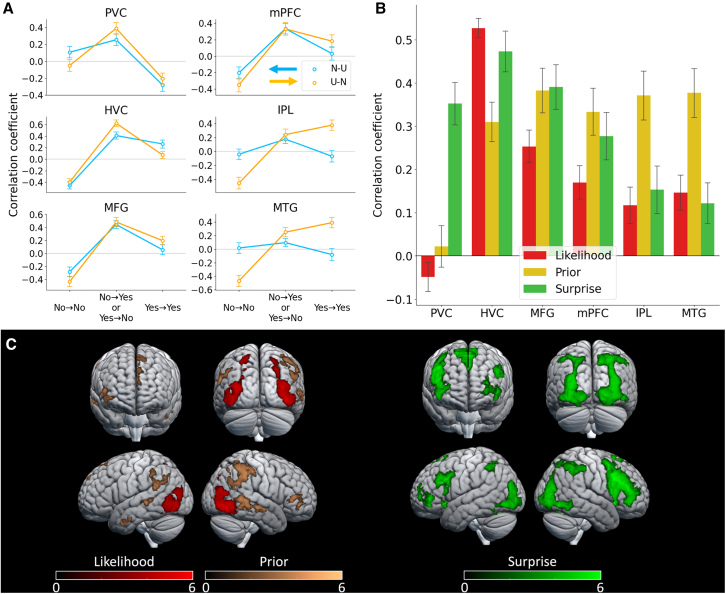
Model-based analysis of fMRI (A) ROI-wise correlation coefficients between the percent signal change at each anatomical ROI (see STAR Methods) and the joint probability (predicted by the Bayesian model, Figure 2C) of two types of behaviors, “Yes” (recognizable) and “No” (un-recognizable), for pairs of previously and currently presented images. Blue and orange represent the N-U and U-N conditions, respectively. Circles indicate the average values across the participants. Error bars indicate the standard error. (B) ROI-wise correlation coefficients between the percentage signal change at each ROI and each of the three model-based indicators: “likelihood” (red), “prior” (yellow), and “surprise” (green). Error bars indicate the standard error of the mean. (C) Results of the parametric modulation analysis (voxel level: *p* < 0.0001, uncorrected; cluster level: *p* < 0.05, FWE-corrected). Brain regions correlated with each of the three model-based indicators: “likelihood” (red), “prior” (yellow, Voxel-level: *p* < 0.001, uncorrected, minimum 100 voxels), and “surprise” (green).

These findings were consistent with the brain imaging analysis of natural and unnatural images presented in a random order during the control condition (Figure S2A). The percentage of signal changes in these areas correlated with the degree of naturalness, irrespective of the presentation order (Figure 3A). Notably, in the MFG, the percent signal change (PSC) occurred with images at the naturalness level of 0.4–0.6 in the N-U condition and 0.6 to 0.8 in the U-N condition. This aligns with participant responses switching more frequently regarding image-scene recognizability (Figure 2B). Specifically, the MFG was activated when the participants’ report was changed (Figure S2B) and exhibited higher correlation coefficients between the PSC and the estimated probabilities of participants’ report changes (Figures 4A and S3A).

### Image presentation order influenced brain activities

When comparing brain activity as a function of the timing of image presentation, the bilateral inferior occipital gyrus (IOG) and the supplementary motor cortex were activated during the first trial of the blocks (Figure 3B, red). In contrast, the medial prefrontal cortex (mPFC, BAs 8/9), inferior parietal lobules (IPL, BAs 39/40), and middle temporal gyrus (MTG, BA21) exhibited increased activity during the last image presentation in the blocks, regardless of the naturalness level of the image (left mPFC: [-12,56,36], right mPFC: [18,58,34], left IPL: [-58,-60,24], right IPL: [56,-38,44], left MTG: [-50,10,-30], right MTG: [50,12,-32], Figure 3B, yellow). Within these ROIs, the correlation between the image naturalness level and the PSC showed different polarities depending on the image presentation order. Specifically, the PSCs in the mPFC, IPL, and MTG decreased when the image naturalness was high in the N-U condition, whereas they increased when the image naturalness was low in the U-N condition (Figure 3B). These results suggest that activities in the mPFC, IPL, and MTG were influenced by *a priori* knowledge of the scene depicted in the series of images rather than the image naturalness level per se.

### Correlations with likelihood, prior, and Bayesian surprise

To evaluate the role of these six ROIs, we performed a correlation analysis between the activation of each ROI and three model variables: (1) likelihood certainty, calculated as the log likelihood, logP(α|Yes); (2) prior certainty, calculated as log prior logP(Yes); and (3) Bayesian surprise,^29^ calculated as the KL divergence between the posterior probability of the response chosen by the participants in the current trial and that in the previous trial, KL(P(Yes|α)‖P(Yes))+KL(1−P(Yes|α)‖1−P(Yes)) (Figures 4B and S3B). The model-based variables demonstrated significant correlations with PSCs in nearly all ROIs, primarily due to their high collinearity. The likelihood certainty displayed a stronger correlation with activities in the HVC than with prior certainty. Conversely, prior certainty exhibited its strongest correlation with activities in the mPFC, IPL, and MTG. Surprise, on the other hand, exhibited a stronger correlation with PSC in the MFG than with the certainty of likelihood and prior.

To further investigate, we conducted a GLM analysis where the image presentation period in each trial was modulated by model-based likelihood certainty, prior certainty, and surprise. This parametric imaging analysis revealed that the bilateral HVC exhibited activity positively correlated with likelihood certainty (Figure 4C, red; Table S3). Simultaneously, the mPFC, IPL, and left MTG displayed activity correlated with prior certainty (Figure 4C, yellow; Table S3). Moreover, it was discovered that brain areas exhibiting BOLD activity correlated with model-based surprise not only included regions associated with likelihood and prior certainty but also extended to the anterior prefrontal cortex (Figure 4C, green; Table S3). As the naturalness of the images was correlated with the visual complexity, that is, the mean power spectrum of the image, we also conducted a GLM analysis, which regressed out the image complexity as a nuisance variable. For the surprise and prior certainty, we confirmed that the results were almost identical to those obtained with the GLM, which did not account for the image complexity. This additional GLM analysis exhibited that the PVC activity was correlated with the image complexity, whereas the likelihood certainty activated the activity in the lateral occipital cortices (Figure S3C).

## Discussion

In this study, we designed sets of images deconstructed to various degrees (Figure 1A) and presented each set in the order of deconstruction (from a natural to an unnatural image, Figure 1B middle) or reconstruction (from an unnatural to a natural image, Figure 1B bottom). The aim was to investigate how the brain represents uncertainties in contextual and sensory information. We propose a Bayesian model to predict participants’ responses to sequentially presented image scene recognition (Figure 2C). Behavioral analyses revealed that participants’ image-scene recognizability depended not only on the presented images but also on the order of image presentation. Furthermore, these hysteretic behaviors were effectively replicated by our proposed model (Figure 2A).

The proposed model utilizes the Bayesian formula to update the probability of the participant responding “Yes” to the current image. Participants were aware that the image set comprised deconstructions of the same image, allowing them to utilize scene information from previously presented images in addition to the currently displayed image, especially when aligned in the order of deconstruction. Previous studies employing Bayesian models explained human behavior with hysteresis in perceptual estimation tasks for orientation^30^ and motion^9^^,^^31^ using dots and lines as visual stimuli. Hysteresis was also observed in letter recognition,^32^ scene recognition,^33^ and face memory tasks.^34^ While some studies have elucidated human object recognition using a model that accumulates evidence over time,^35^ none have applied this model to scene recognition. Our results showcase the potential of a sequential Bayesian estimation framework for understanding human information processing in more complex scene recognition within temporally changing environments.

In the proposed model, the parameter C represents the propensity to respond as recognizable. The smaller the value, the more likely the participant is to evaluate the image as recognizable. λ represents the persistence of prior information for the recognizability of the image scene. The larger the value, the more the participant is influenced by the prior information in evaluating the current images. The introduction of C is considered appropriate because the percentage of images that are evaluated as recognizable varies from participant to participant, even for images with the same naturalness level (Figure S1). The introduction of λ adds a bias to the Bayesian inference, and such models have been used in various studies.^36^^,^^37^^,^^38^^,^^39^ Furthermore, we found that model 1 (with variable λ) performed better than the models without lambda in terms of BIC. Therefore, setting λ individually based on individual behaviors is considered appropriate.

To investigate whether GANSID images can control visual attention, we conducted an eye movement measurement experiment. Comparing the gaze distributions for natural images (α = 1.0) and other images revealed that GANSID images exhibited smaller MSE compared to scrambled images (Figure 2D). This difference was particularly pronounced in the N-U condition. In this condition, since a natural image was presented at the beginning of the block, as the image transformed into a scrambled image that lacks salient regions, eye movements were more strongly influenced by top-down attention, likely resulting in different gaze distributions between natural images and other images. On the other hand, unnatural GANSID images contained salient regions, and these regions were positioned similarly to those in natural images. As a result, the gaze distributions for unnatural and natural images remained similar even in the N-U condition. Furthermore, the smaller differences in gaze distributions between the N-U and U-N conditions for the same images (Figure 2E) suggest that GANSID images were able to control visual attention across both image presentation conditions and across all levels of naturalness.

Univariate brain imaging analysis revealed increased activity in the MFG and HVC, including the extrastriate cortices, fusiform gyri, SPL, and posterior parahippocampal gyri, when participants viewed natural images (Figure 3A). These findings align with our earlier study on visual attention using the original GANSID.^24^ The HVC played a crucial role in integrating these properties for perceiving complex objects,^40^^,^^41^ such as faces and bodies.^42^^,^^43^^,^^44^^,^^45^ Further computational analysis indicated that activity in these brain areas correlated with sensory certainty (Figures 4B and 4C). This association is consistent with prior research^46^^,^^47^^,^^48^^,^^49^^,^^50^ in visual cognition, which has shown a connection between activity in the visual cortex and uncertainty^51^ in sensory information, as well as perceptual prediction errors.^52^

The univariate brain imaging analysis also revealed higher activity in the PVC when unnatural images were presented (Figure 3A), and the model-based analysis showed a weak negative correlation between the PVC activity and “likelihood” (Figure 4B). However, the PVC exhibited heightened responsiveness to fundamental visual properties like edges, orientation,^53^ and colors.^54^ Furthermore, in the parametric modulation analysis that included the complexity features of the images, we found the activity in the PVC correlated with the image complexity (Figure S3C). These findings suggest that the activity of the PVC was influenced more by the complexity of the low-level visual properties rather than the sensory uncertainty.

It is thought that the three brain areas, the parahippocampal place area (PPA), the occipital place area (OPA), and the retrosplenial complex (RSC), play important roles in scene recognition.^55^ In the HVC in which activity was observed when natural images were presented compared to unnatural images (Figure 3A), the PPA and OPA were included, but the RSC was not. It has been suggested that the PPA is involved in the spatial layout of a scene and landmark recognition,^56^^,^^57^ and the OPA encodes the environmental boundaries and local elements of the scene.^58^^,^^59^ On the other hand, the RSC is involved in navigation within a large environment,^60^^,^^61^ so it did not show a significant difference in the RSC activity when comparing natural images and unnatural images. However, the differences in the roles of the three areas are still unclear, so further experiments are needed to clarify the reason.

In this study, we employed a deconstructed image set derived from the same original image presented in two different orders. We observed variations in participants’ recognition of image scenes based on the presentation order, even when the images shared the same level of naturalness. The certainty of prior-level information, contingent upon the recognizability of preceding images, enabled our computational model to account for this hysteresis behavior (Figure 2A). The activities in the mPFC, IPL, and MTG were the primary effects associated with images presented at the end of the blocks (Figure 3B). The percentage of signal change when viewing an image with a lower naturalness level was higher in the N-U condition compared to the U-N condition (Figures 3B and S2C). Since participants were exposed to the last images, they had already seen all other images within the same group, providing them with more prior information than at the beginning. Moreover, when participants viewed an image with a lower naturalness level in the N-U condition, they had more prior information due to having seen a more natural image before the current one. Conversely, when they viewed an image with a low naturalness level in the U-N condition, they had less prior information. Therefore, the differences in brain activity between the N-U condition and the U-N condition were attributed to variations in prior information uncertainty.

Moreover, the model-based analysis revealed a correlation between prior certainty and activities in the mPFC, IPL, and MTG (Figures 4B and 4C), all of which are components of the DMN.^62^ Existing research^15^^,^^16^^,^^63^^,^^64^^,^^65^ suggests the involvement of the DMN in processing prior information or prior knowledge in visual information processing. Gonzalez-Garcia et al.^16^ proposed the DMN as a source of prior information, while Levinson et al.^63^ reported that DMN activation showed less reduction when recognition was driven by prior knowledge compared to spontaneous recognition. Consequently, we infer that prior information in our image recognition task is represented by the DMN, encompassing the mPFC, IPL, and MTG.

We employed Bayesian surprise calculations in our model and observed a positive correlation with various brain regions, including visual areas, SPL, mPFC, and MFG (Figures 4B and 4C). The correlation coefficients between the PSC in these regions and the estimated probabilities of participants’ report changes ($\hat{P}\left(Yes\to No\right)$ or $\hat{P}\left(No\to Yes\right)$) were notably high (Figure 4A). Additionally, these brain regions exhibited activity when participants’ reports on recognizability changed (Figure S2B). The SPL and prefrontal cortex are integral components of the FPN,^66^ influencing surprise and conflict processes.^67^ Evaluation changes were categorized into two types: $\hat{P}\left(Yes\to No\right)$ and $\hat{P}\left(No\to Yes\right)$. However, no significant difference was observed in the brain activities associated with these two categories. We also found that the performance of the model with unified hyperparameters λ outperformed the model with varying hyperparameters in the N-U and U-N conditions, as evidenced by the BIC score (Table S1). This suggests a symmetrical relationship between recognizability and un-recognizability in the task. Previous studies^16^^,^^68^ have indicated the activation of visual areas and the FPN when resolving ambiguity in stimuli. Visalli et al.^20^ reported that FPN activity is modulated by surprises related to temporal expectations. Considering these findings, we conclude that the FPN, encompassing the SPL and MFG, represents Bayesian surprise. This reflects how much information from a given image alters the prior, leading to the formation of a new posterior in our image recognition task.

In this study, we employed a deep machine–learning technique to generate a series of images with varying levels of naturalness. Subsequently, we designed a scene recognition task wherein the generated image stimuli were presented in deconstruction order or reverse order. Our proposed computational model, rooted in incremental Bayesian estimation, accurately replicated the hysteric scene recognition behaviors exhibited by the participants, contingent on the display order of the image series. Brain imaging analyses enabled us to differentiate the regions responsible for uncertainty in sensory-level information (or likelihood) and prior-level information during image scene recognition. Our findings revealed that activities in the HVC were associated with likelihood uncertainty, while activities in the DMN, including the mPFC, IPL, and MTG, were associated with prior uncertainty. This discovery enhances our comprehension of the mechanisms underlying visual perception. Furthermore, the results of this study are anticipated to contribute to the optimal design of advertisements and signs that captivate attention and evoke surprise. Additionally, it may inform the development of interfaces capable of decoding attention and surprise from the brain, fostering connections between machines and humans in both real and artificial visual environments.

### Limitations of the study

In this study, we utilized the images generated by the deep image transformation to manipulate likelihood and prior uncertainty. By defining likelihood and prior information from the parameters included in this image transformation method, we were able to construct a model that accurately replicates the behavior of participants. However, it is difficult to define universal likelihood and prior information in scene recognition, making it difficult to evaluate the validity of the model’s settings.

## Resource availability

### Lead contact

Further information and requests for resources should be directed to and will be fulfilled by the lead contact, Kojiro Hayashi (hayashi.kojiro.65r@st.kyoto-u.ac.jp).

### Materials availability

This study neither used any reagents nor generated new materials.

### Data and code availability

• Behavioral and fMRI data have been deposited at Zenodo and are publicly available as of the date of publication. DOIs are listed in the key resources table.
• The original code for generating the image dataset has been deposited in a GitHub repository. The URL is listed in the key resources table.
• Any additional information required to reanalyze the data reported in this paper is available from the lead contact upon request.

## Acknowledgments

This study was supported by a project (JPNP20006) commissioned by the 10.13039/501100001863New Energy and Industrial Technology Development Organization (NEDO), and partly by 10.13039/501100001691JSPS KAKENHI (22H04998, 23H04676), 10.13039/100009619AMED under grant number JP23wm0625001, and MRC/Versus Arthritis (MR/W027593/1). This study has been delivered through the National Institute for Health and Care Research Oxford Health Biomedical Research Centre.

## Author contributions

Conceptualization, W.Y. and S.I.; methodology, K.H., R.K., K.F., and S.I.; software, K.F.; formal analysis, K.H.; investigation, K.H. and R.K.; resources, K.F.; data curation, K.H.; writing – original draft, K.H.; writing – review and editing, R.K., W.Y., and S.I.; visualization, K.H.; supervision, S.I.; project administration, S.I.; funding acquisition, S.I.

## Declaration of interests

The authors declare no competing interests.

## Declaration of generative AI and AI-assisted technologies in the writing process

During the preparation of this work, the authors used ChatGPT and Grammarly in order to improve readability and language. After using these services, the authors reviewed and edited the content as needed and take full responsibility for the content of the publication.

## STAR★Methods

### Key resources table

**Table:** 

REAGENT or RESOURCE	SOURCE	IDENTIFIER
**Deposited data**		

Behavioral and fMRI data	This study	Zenodo: https://doi.org/10.5281/zenodo.13713615 (Subject0∼14) https://doi.org/10.5281/zenodo.13715445 (Subject15∼29)

**Software and algorithms**		

MATLAB R2021b	The MathWorks	https://www.mathworks.com/products/matlab.html
SPM12	Wellcome Trust Centre for Neuroimaging	https://www.fil.ion.ucl.ac.uk/spm/software/
PsychoPy	Peirce et al.^69^	https://www.psychopy.org/
MRICroGL	NeuroImaging Tools & Resources Collaboratory	https://www.nitrc.org/projects/mricrogl
MarsBaR	Brett et al.^70^	https://sourceforge.net/projects/marsbar/
Code for generating the image dataset	This study	GitHub: https://github.com/Keisuke-Fujimoto/Image_Deconstruction_with_Maintained_Saliency

### Experimental model and study participant details

#### Participants

Thirty-one participants (15 males and 16 females, ages 21-49, Japanese) took part in the fMRI study, and twenty-one different participants (12 males and 9 females, aged 19-32, Japanese) participated in the eye tracking task (Table S4). They had no ocular (color) dysfunctions and normal or corrected-to-normal sight and participated in the experiment after receiving an explanation of the task and giving written informed consent. The experiments were approved by the ethics committees of the Graduate School of Informatics, Kyoto University, Kyoto, Japan (KUIS-EAR-2022-010), and Advanced Telecommunications Research Institute International (ATR), Kyoto, Japan (Approval No. 157). One participant did not complete the fMRI task and was therefore excluded from the analyses. Additionally, one participant in the eye tracking task was excluded from the analysis due to a significant number of missed trials (21.3%).

### Method details

#### Stimuli

The image stimuli utilized in the experiment were generated using a modified GANSID^24^ technique (Figure 1A). The architecture of our image-generation method consists of an encoder, generator, and map generator. The generator was trained to generate the original input image with the latent variable $Z_{\phi }$ encoded from the input image and the map latent variable $Z_{\theta }$. The map generator was pre-trained using a saliency map computed using Itti’s method.^28^

Each artificial image was generated by the generator input of the map latent variable $Z_{\theta }$, and also a linear mixture of the latent variable $Z_{\phi }$ and a random number ε that obeys a standard normal distribution with a mixing ratio α. By varying the value of α in the range of 0 to 1, images with gradually different naturalness were generated according to the α value. Since our original GANSID employed α=0.0 and α=1.0, this image generation method can be seen as an extension of the original one. Fifty-four images from the MS COCO database^71^ served as the original images to generate the image stimuli.

For the Gradual blocks (Figure 1B), a set of six naturalness levels (α=1.0, 0.8, 0.6, 0.4, 0.2, and 0.0) was generated from each of the 36 original images, resulting in a total of 216 images. Additionally, from the remaining 18 images, a pair consisting of a natural image (α=1.0) and an unnatural image (α=0.0) was generated for the Binary blocks.

The sets (or pairs) of generated images used in each block condition were consistent across all participants. The image stimuli were presented using a projector (Canon WUX6000, 1920 × 1200 pixels, 60 Hz frame rate) and a mirror setup focused on a central fixation point at a viewing distance of 1120-1135 mm. The size of the image stimuli on the screen was 574 × 393 pixels, corresponding to 10.37-10.51° of visual angle.

#### Image-scene recognition task

In each trial, a fixation cross was presented for 500 ms, followed by a flashing image stimulus at 2.5 Hz (on for 50 ms, off for 350 ms, total 4,000 ms) on the screen. After the presentation of the image stimulus, participants were asked to indicate whether they could recognize the scene depicted in the image. We told the participants that if they were able to well describe the people, objects, and/or place displayed in the image, report “recognizable (Yes)”, or “not recognizable (No)” otherwise. Image-scene recognition was reported by selecting from two options: green (recognizable) or red (un-recognizable) squares using an MRI-compatible response box. To prevent participants from predicting the positions of these options, they were randomly set for each trial. Participants were required to make their choice within 1500 ms, and no-choice trials were excluded from the behavioral analysis (3.33 ± 1.64%; mean ± 95% confidence interval). Participants were also instructed to maintain fixation on the center of the screen as much as possible. The task was programmed in Python using PsychoPy.^69^

Each session comprised eight blocks, each containing six consecutive trials. Our experimental setup involved two types of block conditions: gradual and binary blocks. In a Gradual block, the primary task, participants viewed six image stimuli, constituting a single image set. This set included one natural image (α = 1.0), one unnatural image (α = 0.0), and four mixed images (α = 0.8, 0.6, 0.4, 0.2) derived from the original image. Two display order conditions existed: in the N-U condition, the stimuli were presented in descending order of naturalness level (Figure 1B, middle), while in the U-N condition, they were presented in ascending order of naturalness level (Figure 1B, bottom). On the contrary, in the Binary block, participants encountered only natural (α = 1.0) and unnatural (α = 0.0) images randomly sampled from different pairs of generated images. The block condition was indicated at the block’s onset (for 1500 ms, Figure 1B). Each participant completed 12 binary blocks and 36 gradual blocks distributed across six sessions, where six gradual blocks were randomly interleaved with two binary blocks.

Here, we refer to the naturalness of the images as sensory information and the information about the context depicted in the image, which could be succeeded by the history of the previously observed images, as prior information. In the N-U condition, as the naturalness level decreased, the sensory information gradually decreased, but the prior information would have remained much because the most natural image was initially observed. In the U-N condition, as the naturalness level increased, the sensory information gradually increased, and the prior information would have also increased to accumulate the sensory information.

In the Binary block, participants reported recognizing the images in 97.53% ± 1.37% of trials (mean ± 95% confidence interval) when natural images were displayed. In contrast, they recognized images in only 2.54% ± 1.83% of trials when unnatural images were presented. This result indicates that natural images with a naturalness level of 1.0 were highly recognizable, while unnatural images with a naturalness level of 0.0 were not recognizable.

#### Eye tracking task

We also measured eye movements when participants were viewing the generated images. We used another set of human participants for this follow-up eye-tracking experiment. Participants were positioned 0.6m from a 23.6-inch monitor (Iiyama ProLite B2409HDS, Mouse Computer, Co. Ltd., Tokyo, Japan) in a dark room. The visual stimuli were presented in 960×1280 pixels and subtended 32.5° of visual angle. Eye movements were recorded at 120 Hz with an LED-based eye tracker (Tobii Pro 465 X3-120, Tobii Technology, Tokyo, Japan).

At the beginning of each session, the five-point eye tracker calibration task was conducted. Each trial was performed in the same procedure as the main scanning task (please refer to Image-scene recognition task in STAR Methods). In each session, half of the eight image sets (two GANSID image sets and two scrambled image sets, each of which consisted of six images; α = 1.0, 0.8, 0.6, 0.4, 0.2, 0.0) were presented in the N-U condition sequence, and the other half were presented in the U-N condition sequence. The image sets were presented in a random order across participants. Participants completed nine sessions (432 trials in total).

The GANSID image sets used in the eye-tracking experiment were identical to those used in the Gradual block of the fMRI task. The scrambled image was created by randomly shuffling a proportion of 1-α of the pixels from the natural image generated by GANSID (α=1.0). The input natural images were distinct from those used in the fMRI task. The image with α=1.0 was the same as the natural image, while the image with α=0.0 was an image with all pixels shuffled.

### Quantification and statistical analysis

#### Bayesian model for image recognition

A Bayesian model was developed to elucidate participants' responses (yes/no) in scene recognition (Figure 2C). We assumed that an image at the naturalness level α follows a normal distribution with a mean of α and a variance of σ. P(α|Yes) denotes the likelihood of scene recognizability in a single block. The right area beyond a threshold C (set independently for each participant in the experiment) was labeled as P(α|Yes). Let $\Phi_{\alpha ,\sigma }$ be the cumulative distribution function of the normal distribution with mean α and standard deviation σ, and they are expressed as follows.

(Equation 1) $$P\left(\alpha |Yes\right)=\Phi_{\alpha ,\sigma }\left(2\alpha -C\right)$$

Next, the posterior probability P(Yes|α) is updated iteratively by a Bayesian formula as images are sequentially presented. This is expressed as:

(Equation 2) $$P\left(Yes|\alpha \right)=\frac{e_{y}}{e_{y}+e_{n}}$$

Where:

$$e_{y}=P{\left(Yes\right)}^{\lambda }P{\left(\alpha |Yes\right)}^{1-\lambda }$$

$$e_{n}={\left(1-P\left(Yes\right)\right)}^{\lambda }{\left(1-P\left(\alpha |Yes\right)\right)}^{1-\lambda }$$

Here, P(Yes) is the prior for the current trial, which corresponds to the posterior probability in the previous trial. The hyperparameter λ, defined for each participant, determines the relative weight given to sensory information (P(α∣Yes)) versus prior information (P(Yes)) in calculating the posterior probability for the current trial.

This model has two hyperparameters: C and λ, and we considered four hyperparameter settings: Model 1, which does not fix any hyperparameters, Model 2, which fixes λ at 0, Model 3 which fixes λ at 1, and Model 4 with varying hyperparameter λ in the N-U and U-N conditions. We utilized the Bayesian Information Criterion (BIC) to compare the four model variants. In this comparison, non-fixed hyperparameters were estimated through a grid search to maximize the marginal likelihood of the behavioral data (Table S1). The standard deviation (σ) of the naturalness of the images remained constant for all images and participants.

Model 1, with non-fixed hyperparameters estimated for each participant, outperformed others in terms of BIC scores and effectively replicated participants’ response transitions. We represented pairs of participant responses to consecutively presented images as either Yes → Yes, Yes → No, No → Yes, or No → No. Given a set of naturalness levels presented to the participant, the Bayesian model could predict the joint probability of each pair of responses: $\hat{P}\left(Yes\to Yes\right)$, $\hat{P}\left(Yes\to No\right)$ , $\hat{P}\left(No\to Yes\right)$, or $\hat{P}\left(No\to No\right)$.

(Equation 3) $$\hat{P}\left(Yes\to Yes\right)=P\left(Yes\right)P\left(Yes|\alpha \right)$$

$$\hat{P}\left(Yes\to No\right)=P\left(Yes\right)\left(1-P\left(Yes|\alpha \right)\right)$$

$$\hat{P}\left(No\to Yes)=(1-P\left(Yes\right)\right)P\left(Yes|\alpha \right)$$

$$\hat{P}\left(No\to No)=(1-P\left(Yes\right)\right)\left(1-P\left(Yes|\alpha \right)\right)$$

#### FMRI acquisition and analysis

Imaging data were acquired using a 3T Siemens MAGNETOM Prisma fit scanner (Siemens Healthcare GmbH, Erlangen, Germany) equipped with a standard 64-channel phased array head coil. Interleaved T2∗-weighted echo-planar images were collected using a multiband echo EPI sequence (TR = 1000ms, TE = 30ms, flip angle = 50°, field of view = 200 × 200), with 66 slices per volume and a voxel size of 2 × 2 × 2.5 mm. Additionally, high-resolution T1-weighted structural images (TR = 2250ms, TE = 3.06ms, voxel size = 1 mm × 1 mm × 1 mm) were obtained through standard MPRAGE sequences.

The imaging analyses were carried out using SPM12 (Welfare Department of Cognitive Neurology, London, UK) and MATLAB (MathWorks Inc., Natick, USA). For each participant, all functional images underwent slice timing correction, realignment, and reslicing to the reference functional volume. These were then co-registered to the individual high-resolution anatomical image, spatially normalized to the standard East Asian Brain template, with a resampled voxel size of 2 × 2 × 2 mm. Subsequently, spatial smoothing was applied using a Gaussian kernel filter (FWHM, 8 mm).

The first-level design matrix of the GLM comprised a constant term, six head-motion parameters as nuisance regressors, and 16 event-related regressors. These regressors modeled various phases, including block-condition instruction, choice, and image presentation. Within the 16 event-related regressors, 14 were dedicated to image presentation. These corresponded to six naturalness levels in the N-U and U-N conditions of the gradual block and two naturalness levels in the binary block. These image presentation regressors were represented as a boxcar function time-locked to the onset of image presentation, spanning 4 seconds in each trial. Other event regressors were modeled as boxcar functions with a duration of 1.5 seconds. All event-related regressors were convolved with a canonical hemodynamic response function, and a high-pass filter (cut-off frequency of 1/128 Hz) was applied. The regressors of interest focused on natural and unnatural image trials in the N-U and U-N conditions. For analysis, individual pairwise contrast images corresponding to the main effects (naturalness: natural images in both of the conditions, versus unnatural images in both of the conditions; presentation timing: natural images in the N-U condition and unnatural images in the N-U condition, versus natural images in the U-N and unnatural images in the U-N condition) were subjected to a second-level group analysis using a one-sided t-test.

The parametric modulation analysis (Figure 4) employed a first-level design matrix comprising a constant term, six head-motion parameters as nuisance regressors, and four event-related regressors that modeled the block-condition instruction, choice, and image-presentation phases per session. Within this framework, two types of image-presentation regressors were established: one set for the trials in the binary block and the first trial in the gradual block, and another for the second to sixth trials in the gradual block. In addition, three parametric modulators—likelihood certainty Equation 4, prior certainty Equation 5, and surprise Equation 6—were incorporated. The first variable was orthogonalized in relation to the third, and the second variable was orthogonalized concerning the first and third variables, using the SPM12 software.

(Equation 4) logP(α|Yes)

(Equation 5) logP(Yes)

(Equation 6) $$P\left(\alpha |Yes\right)\log\frac{P\left(\alpha |Yes\right)}{P\left(Yes\right)}-\left(1-P\left(\alpha |Yes\right)\right)\log\frac{1-P\left(\alpha |Yes\right)}{1-P\left(Yes\right)}$$

The group-level analysis utilized a random-effects approach, employing an anatomically localized cerebral cortex. Statistical thresholds were set at a voxel level of p < 0.0001 (uncorrected) and a cluster level of p < 0.05 (FWE-corrected).

Furthermore, we investigated the effect of low-level visual complexity on brain activity. We applied a two-dimensional Fourier transform to the images and calculated their power spectrum. Each image was first converted to a grayscale image, and the power of each phase and each frequency was calculated. The average of the power of all phases and frequencies was used as the feature of complexity for the image. This feature was negatively correlated with the naturalness level (r=-0.81, Spearman’s rank correlation). We included this feature as a nuisance regressor in the model-based GLM analysis (Figure S3C).

Percentage signal changes were computed through the MarsBaR toolbox (https://marsbar-toolbox.github.io/).^70^ The signal value associated with an event was derived by multiplying the beta value corresponding to the event with the regressor in the GLM analysis. Within a session, the average signal was represented by the beta value of the session’s regressor (1 for scans of sensation and 0 otherwise). The maximum estimate of the signal linked to an event was determined as the signal magnitude in response to that event, divided by the average session value, and then multiplied by 100 to express the rate of signal change.

#### ROI selection

The six ROIs, PVC, HVC, MFG, mPFC, IPL, and MTG, were specific brain regions identified as significant in the ANOVA in Figures 3A and 3B in the manuscript. These regions were potentially associated with image naturalness and variations between the two image presentation orders. We used Freesurfer’s^72^ automatic anatomical labeling algorithm^73^ to define a set of six ROIs with an individual’s T1-weighted structural scan. PVC was defined as the occipital pole in Freesurfer’s labeling ‘aparc.a2009s+aseg.mgz,’^74^ HVC as the fusiform gyrus, MFG as the middle frontal gyrus, mPFC as the superior frontal gyrus, IPL as the combined region of the angular gyrus and the supramarginal gyrus, and MTG is defined as middle temporal gyrus.
