# Supplementary material for: Neural mechanisms in resolving prior and likelihood uncertainty in scene recognition
Source: iScience. 2025 May 13;28(6):112663. doi: 10.1016/j.isci.2025.112663 (PMC12158497; doi:10.1016/j.isci.2025.112663)
Supplement: Document S1. Figures S1–S3 and Tables S1–S4 [file mmc1.pdf]

iScience, Volume *28*

Supplemental information

Neural mechanisms in resolving prior and likelihood uncertainty in scene recognition

Kojiro Hayashi, Risa Katayama, Keisuke Fujimoto, Wako Yoshida, and Shin Ishii

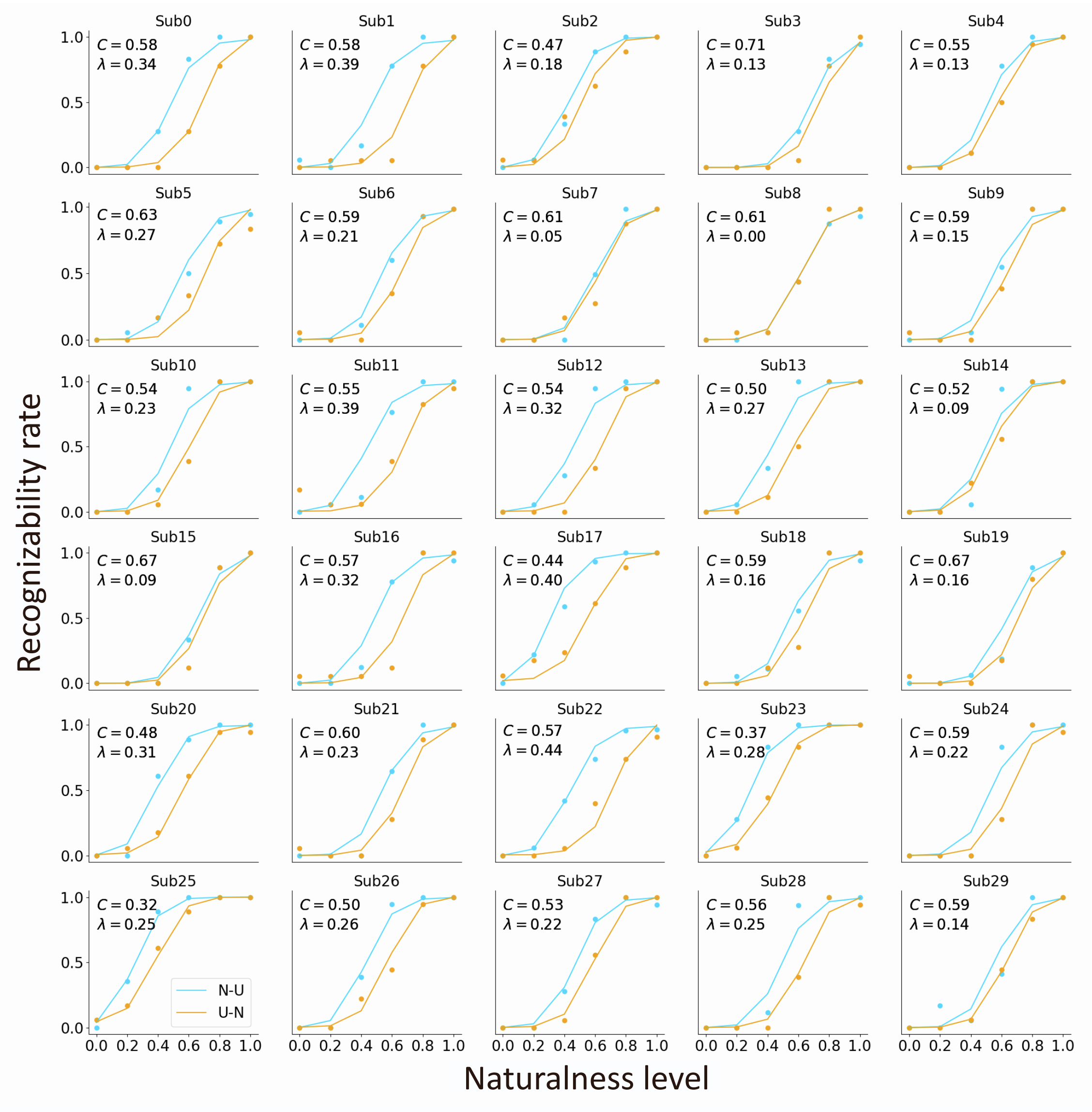

Figure S1. Recognizability rate for all participants, related to Figure 2.

The orange and blue markers correspond to the N-U and U-N conditions, respectively. The solid lines represent the predicted recognizability rates based on the proposed model (Figure 2C). The hyperparameters (λ and C) were estimated for each participant.

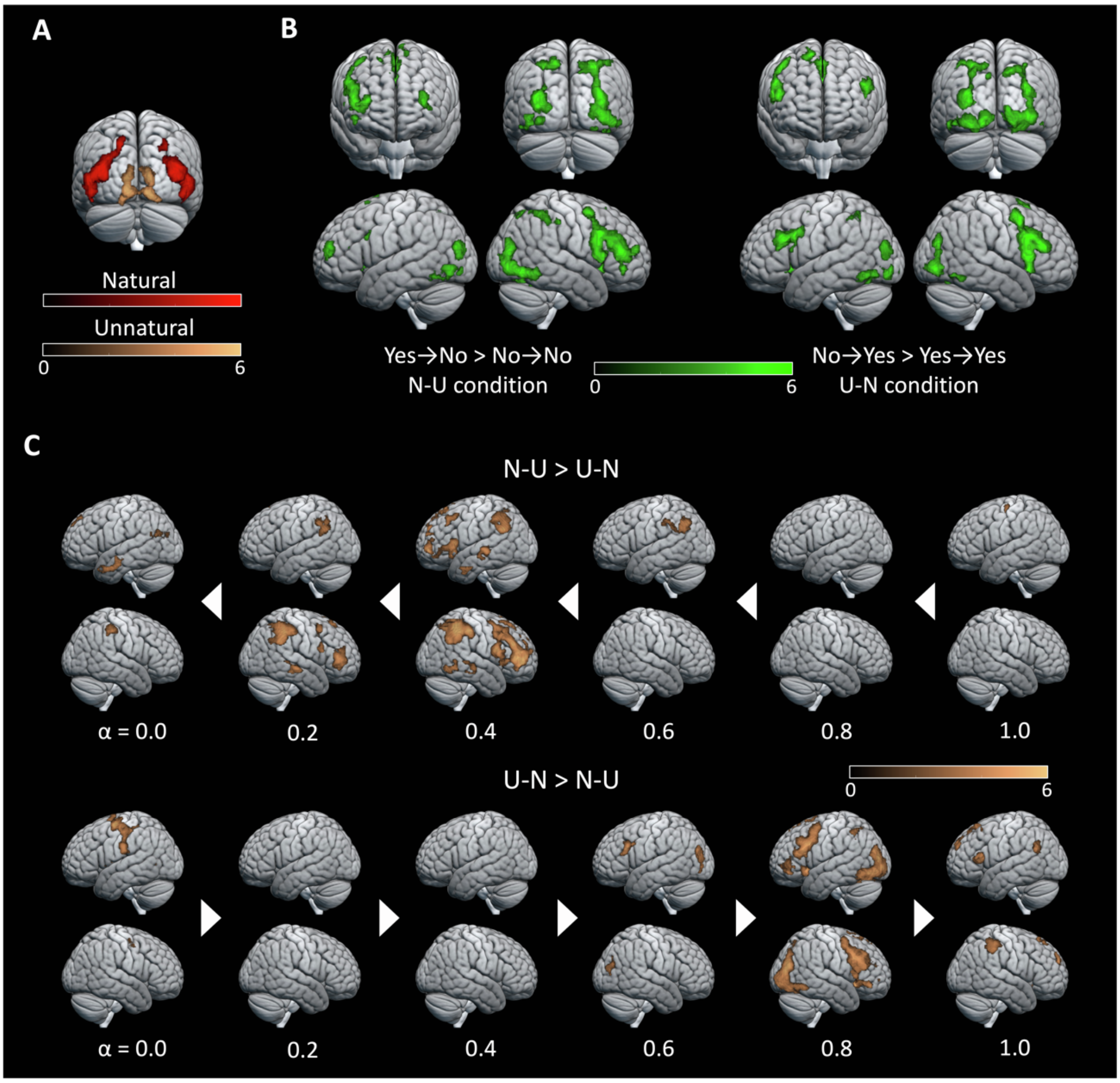

Figure S2. FMRI data analysis (Natural vs Unnatural, Changed vs Unchanged, and N-U vs U-N), related to Figure 3.

(A) Brain Activity When Viewing Natural and Unnatural Images. We examined the brain activity while viewing natural and unnatural images in a binary block. We found that the higher visual cortex was activated when natural images (α=1.0, N) were presented (>unnatural images) and the primary visual cortex was activated when unnatural images (α=0.0, U) were presented (>natural images) (Voxel-level: $p<0.0001$, uncorrected; Cluster-level: $p<0.05$, FWE-corrected). (B) Brain regions were more activated when the participants' responses to two consecutive image presentations were shifted than when they were maintained. (Left) Brain regions that were activated more when the participants' responses were shifted as Yes→No more than they were No→No in the N-U condition. Even when the participants' responses to the currently presented image were the same, the brain activity varied depending on the previous response. Please note that Behavioral shifts in the opposite direction are rare in this condition. (Right)

Brain regions that were activated more when the participants' responses were shifted as No→ Yes more than they were Yes→Yes in the U-N condition (Voxel-level: $p<0.0001$, uncorrected; Cluster-level: $p<0.05$, FWE-corrected).

(C) Differences in brain activity between N-U and U-N conditions. Brain regions activated more in the N-U condition than in the U-N condition (upper panels), and those more in the U-N condition than in the N-U condition (lower panels), for each naturalness level α (voxel level: $p<0.001$, uncorrected; cluster level: $p<0.05$, FWE-corrected).

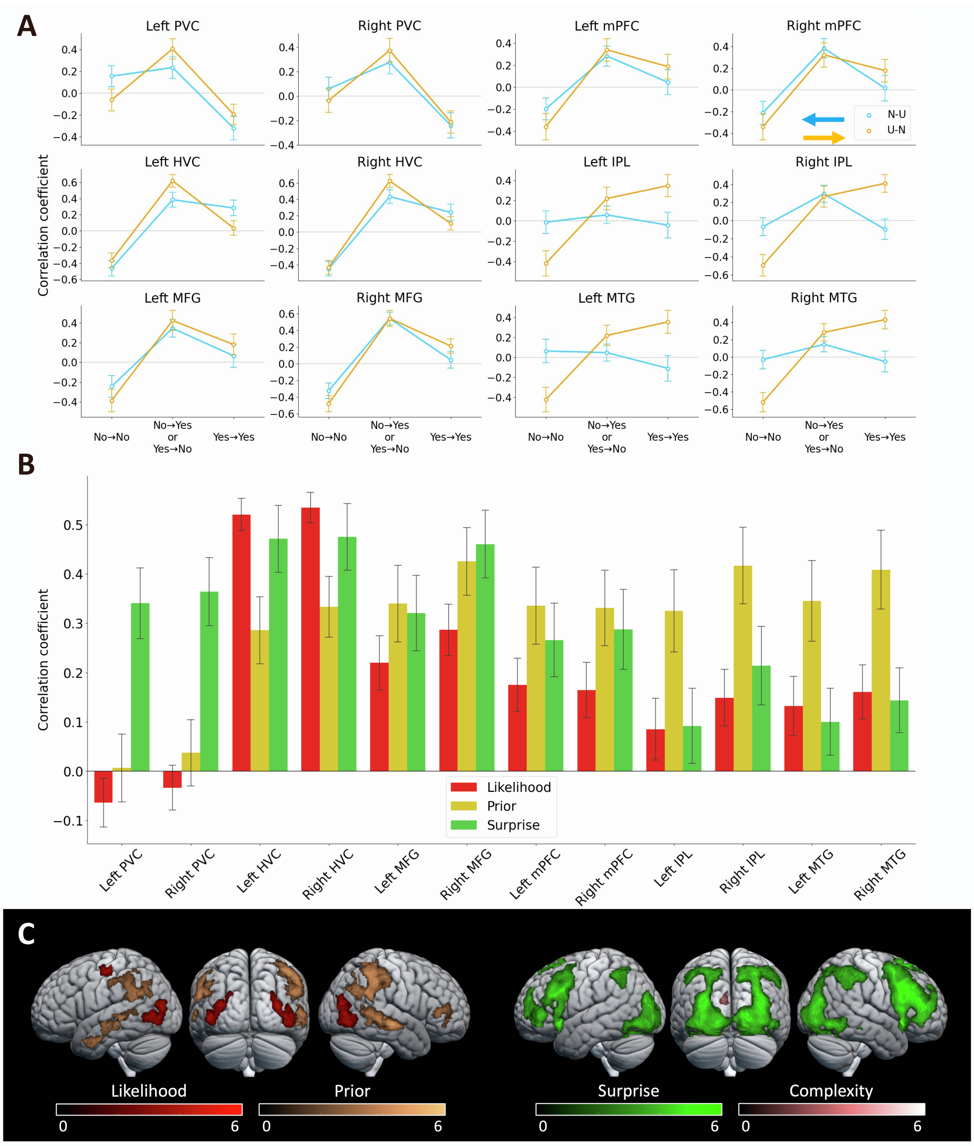

Figure S3. Model-based analysis of fMRI (left and right brain separately), related to Figure 4.

(A) ROI-wise correlation coefficients between the percent signal change at each anatomical ROI (see STAR methods) and the joint probability (predicted by the Bayesian model, Figure 2C) of two types of behaviors, yes (recognizable) and no (un-recognizable), for pairs of previously and currently presented images. Blue and orange represent the N-U and U-N conditions, respectively. Error bars indicate the standard error of the mean.
(B) The ROI-wise correlation coefficient between the percentage signal change at each ROI and each of the three model-based indicators: ‘likelihood’ (red), ‘prior’ (yellow), and ‘surprise’ (green). Error bars indicate the standard error of the mean.
(C) Results of the parametric modulation analysis (voxel level: p<0.001, uncorrected; cluster level: p<0.05, FWE-corrected). Brain regions correlated with each of the four model-based indicators: ‘likelihood’ (red), ‘prior’, ‘surprise’ (green), and ‘complexity’ (pink, Voxel-level: p<0.001, uncorrected, minimum 50 voxels).

Table S1. Bayesian Information Criterion (BIC) scores of the four models with different hyperparameter settings, related to STAR Methods.

	Parameter (mean ± standard deviation)			BIC
	C	λ		
Model1	0.55 ± 0.08	0.23 ± 0.11		3442
Model2	0.56 ± 0.09	-		3485
Model3	0.14 ± 0.01			11881
Model4	0.52±0.14	0.17±0.15 (N-U)	0.30±0.19 (U-N)	3632

Table S2. Peak voxels in areas exhibiting significant activity induced by natural, unnatural, first, and last presented images in the block, related to Figure 3.

Brain region	L/R	MNI coordinates			z-value	No. of voxels
		x	y	z		
Natural image						
Higher visual cortex	L	-38	-82	20	6.28	4927
Higher visual cortex	R	46	-68	-12	6.63	5944
Middle frontal gyrus	L	-44	14	28	4.53	130
Middle frontal gyrus	R	42	14	30	4.96	529
Thalamus proper	L	18	-32	0	6.4	135
Thalamus proper	R	-16	-32	0	5.54	77

Pallidum	R	24	-6	-10	4.54	119
Unnatural image						
Primary visual cortex	L	-2	-90	-10	5.83	400
Primary visual cortex	R	14	-96	10	5.17	168
First image of the block						
Supplementary motor cortex	L/R	-6	12	46	7.16	1689
Inferior occipital gyrus	L/R	-28	-78	-2	6.44	1742
Precentral gyrus	L	-30	-6	56	6.09	1275
Anterior insula	L	-32	18	14	5.48	146
Central operculum	L	-44	-2	12	7.05	145
Parietal operculum	L	-36	-22	18	4.98	142
Precuneus	L	-8	-74	44	5.04	120
Last image of the block						
Medial prefrontal cortex	L	-12	56	36	4.96	507
Medial prefrontal cortex	R	18	58	34	4.88	74
Middle temporal gyrus	L	-50	10	-30	4.25	67
Middle temporal gyrus	R	50	12	-32	4.51	50
Inferior parietal lobule	L	-58	-60	24	4.56	95
Inferior parietal lobule	R	56	-38	44	5.08	438
Superior temporal gyrus	L	-50	-12	-16	4.32	80
Middle frontal gyrus	L	-28	22	56	4.4	74
Middle frontal gyrus	R	30	24	56	4.4	74
Middle frontal gyrus	R	46	42	10	4.71	48

Table S3. Peak voxels in brain regions correlated with each of the three model-based indicators: 'likelihood', 'prior', and 'surprise,' related to Figure 4.

Brain region	L/R	MNI coordinates			z-value	No. of voxels
		x	y	z		
Likelihood						
Higher visual cortex	L	-40	-52	-16	5.14	1608
Higher visual cortex	R	46	-76	-8	6.32	2469

Superior parietal gyrus	L	-26	-60	50	4.72	530
Prior						
Medial prefrontal cortex	L	-2	62	24	4.07	102
Medial prefrontal cortex	R	-12	28	56	3.66	113
Inferior parietal lobule	L	-62	-50	26	4.71	375
Inferior parietal lobule	R	54	-38	44	4.60	1394
Middle temporal gyrus	L	-66	-34	-2	3.78	122
Middle temporal gyrus	L	-46	-2	-30	3.71	113
Middle temporal gyrus	R	52	-18	-10	4.39	1073
Middle frontal gyrus	R	30	42	2	4.14	379
Amygdala	R	30	2	-16	4.35	316
Surprise						
Occipital gyrus	R	28	-48	46	6.73	6053
Occipital gyrus	L	-36	-86	26	6.09	4322
Middle frontal gyrus	L	-28	26	2	6.14	1966
Middle frontal gyrus	R	28	24	0	6.39	4969
Medial prefrontal cortex	L/R	4	30	44	6.53	2685
Inferior frontal gyrus	L	-48	46	0	5.41	518
Caudate	R	16	2	18	5.67	895
Orbital gyrus	R	24	38	-16	5.67	193

Table S4. Details of the participants, related to STAR Methods.

MRI task			Eye tracking task		
No.	Age	Gender	No.	Age	Gender
sub0	24	F	sub0	20	F
sub1	25	F	sub1	19	F
sub2	26	M	sub2	25	F
sub3	38	F	sub3	23	F
sub4	48	F	sub4	19	M
sub5	26	M	sub5	22	M
sub6	45	F	sub6	22	F

sub7	36	F	sub7	24	M
sub8	25	M	sub8	21	M
sub9	37	M	sub9	19	M
sub10	22	M	sub10	28	F
sub11	22	M	sub11	32	M
sub12	40	F	sub12	20	M
sub13	48	F	sub13	24	M
sub14	39	M	sub14	22	M
sub15	21	M	sub15	20	F
sub16	47	F	sub16	21	F
sub17	21	M	sub17	24	M
sub18	36	M	sub18	25	M
sub19	25	F	sub19	24	F
sub20	23	M	(Sub 20)	20	M
sub21	22	F			
sub22	36	F			
sub23	31	M			
sub24	24	M			
sub25	21	F			
sub26	21	F			
sub27	24	M			
sub28	49	F			
sub29	22	F			
(Sub30)	23	M			
